# Analysis of fungal diversity in processed jujube products and the production of mycotoxins by typical toxigenic *Aspergillus* spp.

**DOI:** 10.3389/fmicb.2025.1499686

**Published:** 2025-03-26

**Authors:** Tianzhi Li, Hua Ji, Jingtao Sun, Yinghao Li, Yue Xu, Wenyi Ma, Han Sun

**Affiliations:** ^1^Key Laboratory of Agricultural Product Processing and Quality Control of Specialty (Co-construction by Ministry and Province), School of Food Science and Technology, Shihezi University, Shihezi, Xinjiang, China; ^2^Key Laboratory for Food Nutrition and Safety Control of Xinjiang Production and Construction Corps, School of Food Science and Technology, Shihezi University, Shihezi, Xinjiang, China; ^3^Engineering Research Center of Storage and Processing of Xinjiang Characteristic Fruits and Vegetables, Ministry of Education, School of Food Science and Technology, Shihezi University, Shihezi, Xinjiang, China

**Keywords:** processed jujube products, fungi, mycotoxins, aflatoxin, ochratoxin

## Abstract

Processed jujube products are susceptible to contamination by fungi such as *Aspergillus* spp., which produces mycotoxins that could lead to health problems in consumers. In this study, 58 samples of processed jujube products (including 5 types such as dried jujubes) were collected from different markets in Shihezi (Xinjiang, China). The fungal diversity and the fungi isolated from processed jujube products were systematically analyzed through high-throughput sequencing and molecular biological identification (based on the ITS and/or *BenA* and *CaM* regions). In total, the 105 strains of fungi were isolated and identified as belonging to the dominant genera were *Aspergillus*, *Cladosporium*, *Alternaria*, and *Penicillium*. High-throughput sequencing indicated that *Alternaria*, *Didymella*, *Cladosporium*, and *Aspergillus* were the dominant fungi in processed jujube products. ELISA showed that *A. flavus* produced about 19.3862–21.7583 μg/L, 6.5309–11.0411 μg/L, 0–15.4407 μg/L, 0–5.6354 μg/L, and 0–6.0545 μg/L of AFT, AFB_1_, AFB_2_, AFM_1_, and AFM_2_, respectively. In addition, concentrations of OTA produced by *A. niger, A. tubingensis,* and *A. ochraceus* were found to range from 5.2019 to 18.5207 μg/L. Therefore, the separation of *Aspergillus* with good mycotoxin-producing abilities from processed jujube products poses a latent threat to consumer health.

## 1 Introduction

Jujube (*Zizyphus jujuba* Mill.), belongs to the Rhamnaceae family and has been cultivated for 2000 years in Xinjiang province and more than 4,000 years in China overall (Aafi et al., 2022; Yuan et al., 2019; Cheng et al., 2020). China is the largest producer of jujube and accounts for more than 90% of the world’s jujube production (Liu et al., 2022). Due to their high water content, fresh jujubes are perishable and susceptible to fungal contamination during harvesting, processing, transportation, and storage (Zhang et al., 2022). The common fungal genera that contaminate jujubes are *Aspergillus, Alternaria, Penicillium,* and *Fusarium*, among others (Wu et al., 2017; Bertuzzi et al., 2015; Zhou et al., 2021). Xu et al. (2023) investigated that the main fungi present in decaying red jujubes were *Fusarium* and *Alternaria*. Meanwhile, Xin et al. (2023) reported that red jujubes were particularly susceptible to contamination with *A. flavus*, *A. ochraceus*, *A. niger*, and *Alternaria*. In addition to directly causing pathological effects in plants, fungal contamination also leads to the production of mycotoxins (Kebede et al., 2020; James and Zikankuba, 2018). Furthermore, fungi and mycotoxins from the raw materials could enter the product (Schabo et al., 2021). Therefore, mycotoxin contamination is a major safety concern among consumers who purchase processed jujube products (Mukhtar et al., 2023).

Most of the jujube produced is processed by drying and frying to reduce its moisture content and prolong its shelf life (Bi and Chen, 2016). However, mycotoxin production is not easily degraded without special treatment, and processing could not completely remove mycotoxins (Drusch and Ragab, 2003). Given that processed jujube products are typically sold in a ready-to-eat form, they could lead to mycotoxin exposure if they contain mycotoxins, causing adverse effects on human health. Notably, mycotoxins are toxic secondary metabolites produced by fungi during their growth and reproduction (Yang et al., 2020; Janik et al., 2020). Common mycotoxins include aflatoxins, ochratoxins and *Alternaria* toxins. In particular, AFT (Aflatoxin) and ochratoxins are currently considered the most threatening of all mycotoxins (González-Curbelo and Kabak, 2023). Azaiez et al. (2015) showed that the 83% of all jujube samples were found to be contaminated with at least one mycotoxin, of which 23% contained aflatoxin and 22% ochratoxin A. Iqbal et al. (2014) found that the 31.6% of the jujube products were contaminated by aflatoxin, of which 16 samples exceeded the AFB_1_ (aflatoxin B_1_) content, and 20 samples exceeded the total aflatoxin content. Specifically, mycotoxins are known to have a negative impact on human health, exerting adverse effects such as hepatotoxicity, genotoxicity, and mutagenicity (Stoev, 2021; Cao et al., 2022; Mujahid et al., 2020; Puntscher et al., 2018).

In this context, the aim of the present study was to: (a) isolate representative toxin-producing fungi from samples of processed jujube products sold in the Shihezi (Xinjiang, China) wholesale markets; (b) identify and analyze the fungal species present in processed jujube products at the molecular level and to analyze the diversity of fungi in processed jujube products; and (c) analyze the metabolic features of typical toxin-producing *Aspergillus* strains (*A. flavus*, *A. niger*, A*. tubingensis*, and *A. ochraceus*) and identify typical secondary fungal metabolites that might be present in processed jujube products.

## 2 Materials and methods

### 2.1 Sample

From April to May 2023, a total of 58 processed jujube product samples [5 samples of dried jujube samples (ZG), 17 samples of jujube kernel cake samples (ZRG), 5 samples of crispy jujube samples (CZ), 19 samples of milk jujube samples (NZ), and 12 samples of jujube with walnut samples (ZJHT)] were collected from different markets in Shihezi City (Xinjiang, China). The average weight of each sample was more than 500 g. Among the processed jujube products, the ZG and the CZ were packed in bulk, and the ZRG, the ZJHT, and the NZ were vacuum packed. The sampling methods utilized ensured representative sampling of a wide range of markets in the region. The collected samples were sealed (relative humidity 39%) and stored in a refrigerator at −20°C to prevent fungal growth and metabolite production. After analysis and data evaluation, all samples were treated appropriately.

### 2.2 Chemicals and reagents

Potato dextrose water (PDW) medium, Bengal red agar medium, potato dextrose agar (PDA) medium, toxin-producing medium, Tris–Hcl, ethylenediaminetetraacetic acid, and boric acid were purchased from Qingdao HaiBo Biotech Co., Ltd. (Shandong, China). The Biospin fungal genomic DNA extraction kit was purchased from Hangzhou Bori Technology Co., Ltd. (Zhejiang, China). The 6 × loading buffer, dNTPs, 10 × buffer, Tap DNA polymerase, DNA maker, and Gold View nucleic acid dyes were purchased from Tiangen Biochemical Technology Co., Ltd. (Beijing, China). Agarose was purchased from Biowest (Spain); *A. flavus*, *A. niger*, *A. tubingensis*, and *A. ochraceus* toxin-producing genes primers were purchased from Sangon Bioengineering Co., Ltd. (Shanghai, China). Enzyme-linked immunosorbent assays (ELISA) detection kits for the AFT, AFB_1_, AFB_2_ (aflatoxin B_2_), AFM_1_ (aflatoxin M_1_), AFM_2_ (aflatoxin M_2_), and OTA (ochratoxin A) were purchased from Jiangsu Jingmei Biotechnology Co., Ltd. (Jiangsu, China).

### 2.3 Determination and analysis of fungal diversity

The 58 samples of processed jujube products were sent to Shanghai Meiji Biopharmaceutical Technology Co., Ltd. for high-throughput sequencing in order to analyze their fungal diversity, with each sample tested in six replicates. Sequencing yielded double-terminal paired-end (PE) reads. Firstly, the double-terminal PE reads were assembled into PE double-terminal sequences using FLASH version 1.2.11^1^ while simultaneously ensuring quality control and filtering sequences that do not meet quality criteria. Based on a 97% threshold of sequence similarity, the sequences were clustered into operational taxonomic units (OTUs) using UPARSE (http://www.drive5.com/uparse/ dint version 11). In addition, the tool may also be utilized for identifying and removing chimeric sequences. Each representative OTU sequence was taxonomically annotated based on the ITS database (http://unite.ut.ee/index.php dome Unite 8.0) using RDP Classifier (https://sourceforge.net/projects/rdp-classifier/, version 2.13) and a 0.7 confidence threshold. Taxonomic classifications at the phylum, class, order, family, genus, and species levels were analyzed and plotted with QIIME (http://qiime.org/install/index.html, version 1.9.1). R software (version 3.3.1) was used to run dilution curve analyses, Venn diagram analyses, and community composition analyses (bar and pie diagrams). This software was also used to plot a heat map of the relationship between dominant fungal genera and fruit quality (package pheatmap 1.0.8), as well as to conduct principal coordinate analysis (PCoA) and statistical analysis.

### 2.4 Determination of the total number of fungal colonies, water activity, and pH of processed jujube products

A total of 58 processed jujube product samples were collected. Each sample was placed in aseptic saline and homogenized to obtain a homogenized solution. This solution was subjected to serial dilutions. Approximately 2–3 suitable gradients were selected. Then, the fungal solution was added to the PDA medium, mixed, and cultured (Ma, 2018). Three parallel experiments were performed for each dilution gradient.

Firstly, the sample from each category were obtained. Then, fresh distilled water was added to a beaker containing the samples and heated in a water bath at 50°C for 30 min. The samples were homogenized until the sample solution became uniform. After that, the pH value of the processed jujube products was subsequently measured (Shen and Hui, 1989). For water activity analysis, each sample was placed into a sample dish. After the instrument was fully balanced, the response value was recorded using a water activity meter (Institute HQaTSaI, 2016). Three parallel measurements were obtained for each type of product.

### 2.5 Fungal isolation

Each sample was randomly weighed (50 g, accounting for 10% of the total sample). The samples were cultured in a PDW medium at 28°C for 16 h, and the non-sample PDW medium was set as the control. About 1,000 μL of the enrichment solution and the blank control were pipetted onto the Bengal red agar medium, spread evenly with a sterile spreading rod, and incubated in a constant-temperature biochemical incubator at 28°C for 5 days. Colonies in each sample plate were observed, purified into a single colony using the plate marking method, and subsequently cultured (Guo and Ji, 2021).

### 2.6 Fungal identification

Fungal tissue from the isolated and purified single colonies was ground into fine powder in liquid nitrogen. According to the instructions, the Biospin fungal genomic DNA extraction kit was used to extract genomic DNA. Internal transcribed spacer region (ITS) and/or *β*-microtubulin (*BenA*) and calmodulin (*CaM*) gene sequences were used to analyze the phylogeny of *Aspergillus* (Susca et al., 2020; Deng et al., 2021; Guo et al., 2016). The PCR detection primers are indicated in Supplementary Table S1. Fungal characteristics and genera and species classifications were determined by PCR amplification and sequencing. The PCR reaction system (with a final volume of 25 μL) consisted of 12.5 μL PCR TaqMix (A solution composed of 6 × loading buffer, dNTPs, Tap DNA polymerase and other components), 1 μL primer 1, 1 μL primer 2, 0.5 μL genomic DNA, and 10 μL ddH_2_O. The PCR amplification program was performed under the following conditions: pre-denaturation (94°C, 4 min); 30 cycles of denaturation (94°C, 30s), annealing (55°C, 1 min), and elongation (72°C, 1 min); and the final extension (72°C, 10 min) (Hu et al., 2022; Okayo et al., 2020). The PCR products were electrophoresed with 1% agarose gel in 1 × TBE electrophoresis buffer (A buffer solution composed of Tris–Hcl, ethylenediaminetetraacetic acid, and boric acid). Latterly, the electrophoresis results were detected using the gel imaging system to determine whether the amplification was successful. Subsequently, the PCR products were subsequently sent to Youkang Bioengineering (Xinjiang) Co., Ltd. for sequencing. The consistency sequence was checked by the View Sequencer file (Trace Editor) from MEGA 7.0 software, and the Basic Local Alignment Search Tool (BLAST) was used for comparison and analysis. Phylogenetic analysis also was carried out using MEGA 7.0 software. After linked sequence alignment, nucleotide gaps and missing data were deleted. Each phylogenetic tree was built using the neighbor connection method.

### 2.7 Detection of the toxin-producing genes of typical *Aspergillus* in processed jujube products

The four typical toxigenic *Aspergillus* (*A. flavus, A. niger, A. tubingensis*, and *A. ochraceus*) were isolated from processed jujube products. The aflatoxin-producing and ochratoxin A-producing genes from NCBI-GenBank were selected. The seven aflatoxin-producing genes (*aflD*, *aflP*, *alfQ*, *aflR*, *aflS*, *omtA*, *ver*) and seven ochratoxin A-producing genes (*AcLaeA*, *otaA*, *otaB*, *otaC*, *otaD*, *pks*, *nrps*) of the obtained isolates were detected using PCR (Akinola et al., 2019; Więckowska et al., 2023; Maor et al., 2021; Quaglia et al., 2020). The primers targeting the toxin-producing genes used for PCR analysis are shown in Supplementary Table S2. The *BenA* gene sequence served as a positive control for the genome and PCR conditions. Based on the amplification of the above mentioned toxin-producing genes, it was determined whether a strain produced a particular toxin. If the amplification results were negative, the strain was considered to not produce the toxin. If the amplification results were positive, it indicated the presence of toxin-producing genes in the strain, but it was still necessary to use the ELISA to further analyze whether these strains produce toxins.

### 2.8 Cultivation and toxin detection of typical toxigenic *Aspergillus*

The toxin-producing *A. flavus, A. niger, A. tubingensis*, and *A. ochraceus* strains were isolated. From each of above strain cultures, three mycelial plug (3 mm diameter) was taken to inoculate into 50 mL of toxin-producing medium. After 10 days of incubation in the dark, on an orbital shaker at 130 r/min and at 22 ± 2°C, each fungal culture was filtered through sterile filter paper to separate mycelia and culture filtrate. Subsequently, the culture filtrate was centrifuged at 8000 r/min, the supernatant was taken, and stored at −80°C (Okayo et al., 2020). Employing the ELISA method to measure the contents of AFT, AFB_1_, AFB_2_, AFM_1_, AFM_2_, and OTA in the supernatant from single-strain culture filtrate. The toxin content in each sample was measured in triplicate. The linear range of AFT is 0.1–25 μg/L, and the linear range of AFB_1_, AFB_2_, AFM_1_, AFM_2_, and OTA is 0.1–80 μg/L. The linear relationship is known to be well-characterized in this range (R^2^>0.997, the average recovery rate: 96.36–108.08%).

### 2.9 Statistical data analysis

The obtained sequences were compared using BLAST in the NCBI database. Gene sequences with higher homology were selected, and MEGA 7.0 software was used to construct a phylogenetic tree using the neighbor-joining method. Mycotoxin content was analyzed by Microsoft Excel 2020 software. The sample group significance of water activity, pH value, and total colony count was analyzed by SPSS 20.

## 3 Results

### 3.1 Total number of fungal colonies, water activity, and pH of processed jujube products

The total number of fungal colonies, water activity, and pH value of the processed jujube products are displayed in Tables 1, 2. The range of the logarithmic total colony counts for commercially available processed jujube products is from 1.26 ± 0.19–2.01 ± 0.053 log cfu/g. Particularly, the sample in ZJHT had the highest colony count (2.01 ± 0.053 log CFU/g). In general, the standard for the total number of fungal colonies in processed jujube products is not to exceed 150 CFU/g (The logarithmic value – 2.17) (Association HFI, 2020). The above results showed that all samples were contaminated with microorganisms, but all of them were below the standard requirements for date products mentioned above.

**Table 1:** The total number of fungal colonies in different processed jujube products.

Product name	Mean fungal colony counts (log CFU/g) ± S.D.	Range (CFU/g)
ZG	1.560 ± 0.076^d^	26–46
ZRG	1.770 ± 0.064^b^	36–78
CZ	1.260 ± 0.190^e^	9–37
NZ	1.750 ± 0.098^c^	30–83
ZJHT	2.010 ± 0.053^a^	77–129

S.D., standard deviation; ZG represents dried jujube; ZRG represents jujube kernel cake; CZ represents crisp jujube; NZ represents milk date; ZJHT represents jujube with walnut; ^abcde^ represents different significance, *p* < 0.05.

**Table 2:** The water activity and pH value in different processed jujube products.

Product name	Water activity ± S.D.	Range	pH value ± S.D.	Range
ZG	0.3060 ± 0.0010^e^	0.3020–0.3090	5.460 ± 0.025^b^	5.430–5.520
ZRG	0.5350 ± 0.0015^c^	0.5310–0.5390	5.230 ± 0.010^c^	5.210–5.260
CZ	0.3080 ± 0.0006^d^	0.3050–0.3100	5.620 ± 0.015^a^	5.590–5.650
NZ	0.6310 ± 0.0020^a^	0.6260–0.6360	5.270 ± 0.025^c^	5.230–5.340
ZJHT	0.6130 ± 0.0012^b^	0.6110–0.6160	4.480 ± 0.012^d^	4.460–4.520

S.D., standard deviation; ZG represents dried jujube; ZRG represents jujube kernel cake; CZ represents crisp jujube; NZ represents milk date; ZJHT represents jujube with walnut; ^abcde^ represents different significance, *p* < 0.05.

In the product samples, the pH value ranges from 4 to 6. This result indicates that the product samples are weakly acidic, which is suitable for the growth of most fungi. The water activity ranges from 0.30 to 0.65. with the CZ sample being the lowest (0.308 ± 0.0006) and the NZ sample being the highest (0.631 ± 0.0020). It is worth paying attention that although the ZG and CZ samples are bulk, they have lower water activity, resulting in fewer isolated and purified fungi (1.56 log CFU/g and 1.26 log CFU/g, respectively). In contrast, the ZRG and ZJHT samples have higher water activity and therefore contain more fungi (1.77 log CFU/g and 2.01 log CFU/g, respectively). From the above results, it could be concluded that high water activity is more favorable for fungal growth. The above findings are similar to the results obtained from the colony counts.

### 3.2 Fungal diversity analysis of processed jujube products

High-throughput sequencing was used for the analysis of fungal diversity in processed jujube products samples. A total of 4,645,507 reads were obtained from the 58 product samples, and the effective fungal sequence length was found to be 211–300 bp. The optimized fungal sequences were clustered into OTUs based on a similarity threshold of 97%, and 1,012 OTUs in total of were obtained. The number of OTUs in each sample was found to be between 208 and 450. The coverage rate of the samples was determined to be greater than 99%, indicating that the current sequencing volume provided an appropriate representation of the composition of the fungal communities present in processed jujube products.

**Figure 1:**
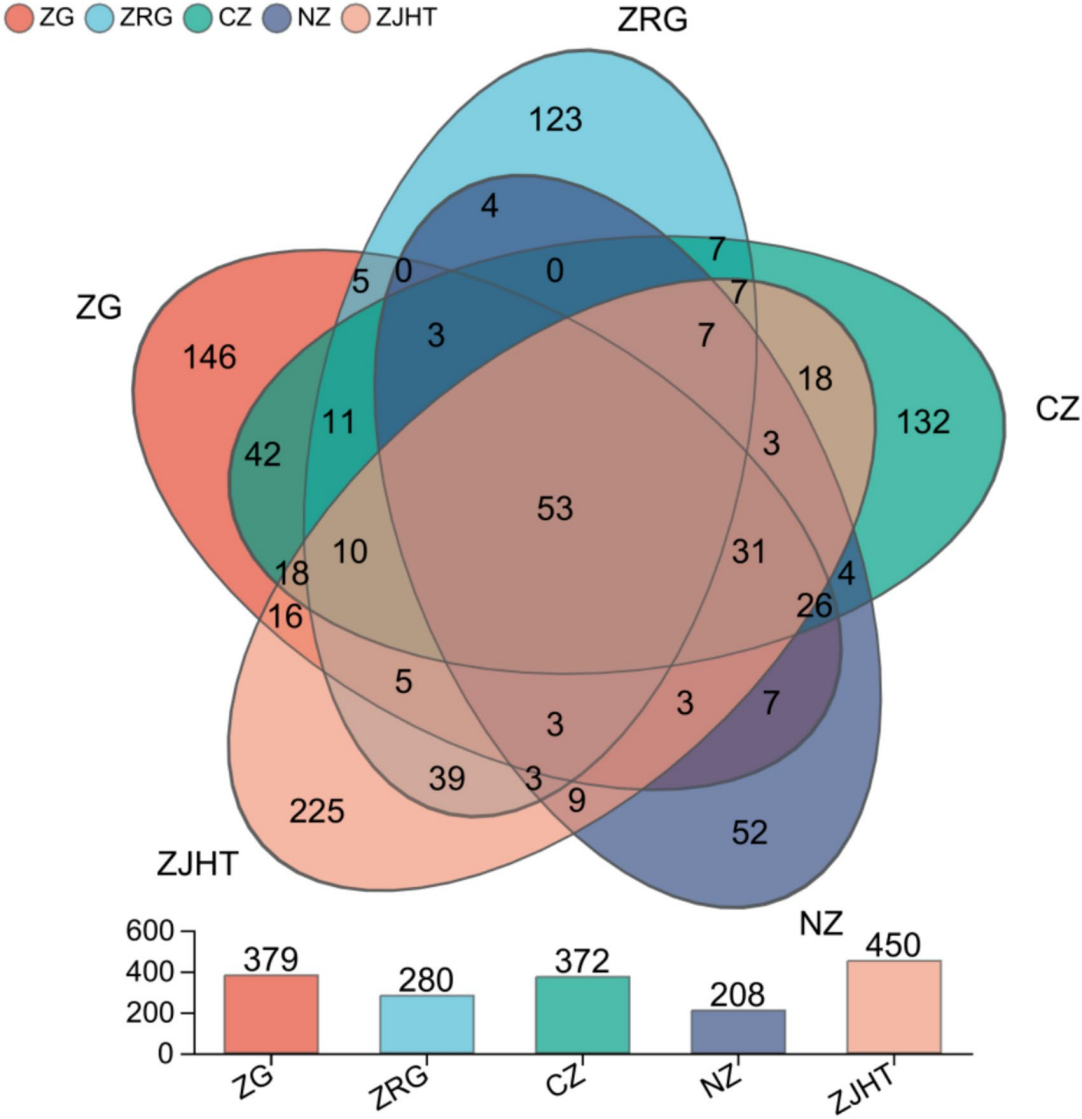
Venn diagram of fungal communities. ZG represents dried jujube; ZRG represents jujube kernel cake; CZ represents crisp jujube; NZ represents milk date; ZJHT represents jujube with walnut.

In the samples of processed jujube products, all fungal OTUs could be divided into 11 phyla, 37 classes, 85 orders, 206 families, and 392 genera. The results are shown in Figure 1. Among the 1,012 OTUs, 53 OTUs were found to be common across all samples and were considered the core fungal microorganisms of processed jujube product samples. The number of unique fungal OTUs for the ZG, the ZRG, the CZ, the NZ, and the ZJHT were 146, 123, 132, 52, and 225, respectively. Based on the results, it could be analyzed that the fungal community in the ZJHT exhibits greater diversity. Further analysis revealed that the 53 core fungal groups belonged to the *Ascomycota* and *Basidiomycota* phyla mainly comprising *Didymella* (20.59%), *Alternaria* (14.96%), *Cladosporium* (10.71%), and *Aspergillus* (7.39%) (Supplementary Figure S3).

**Figure 2:**
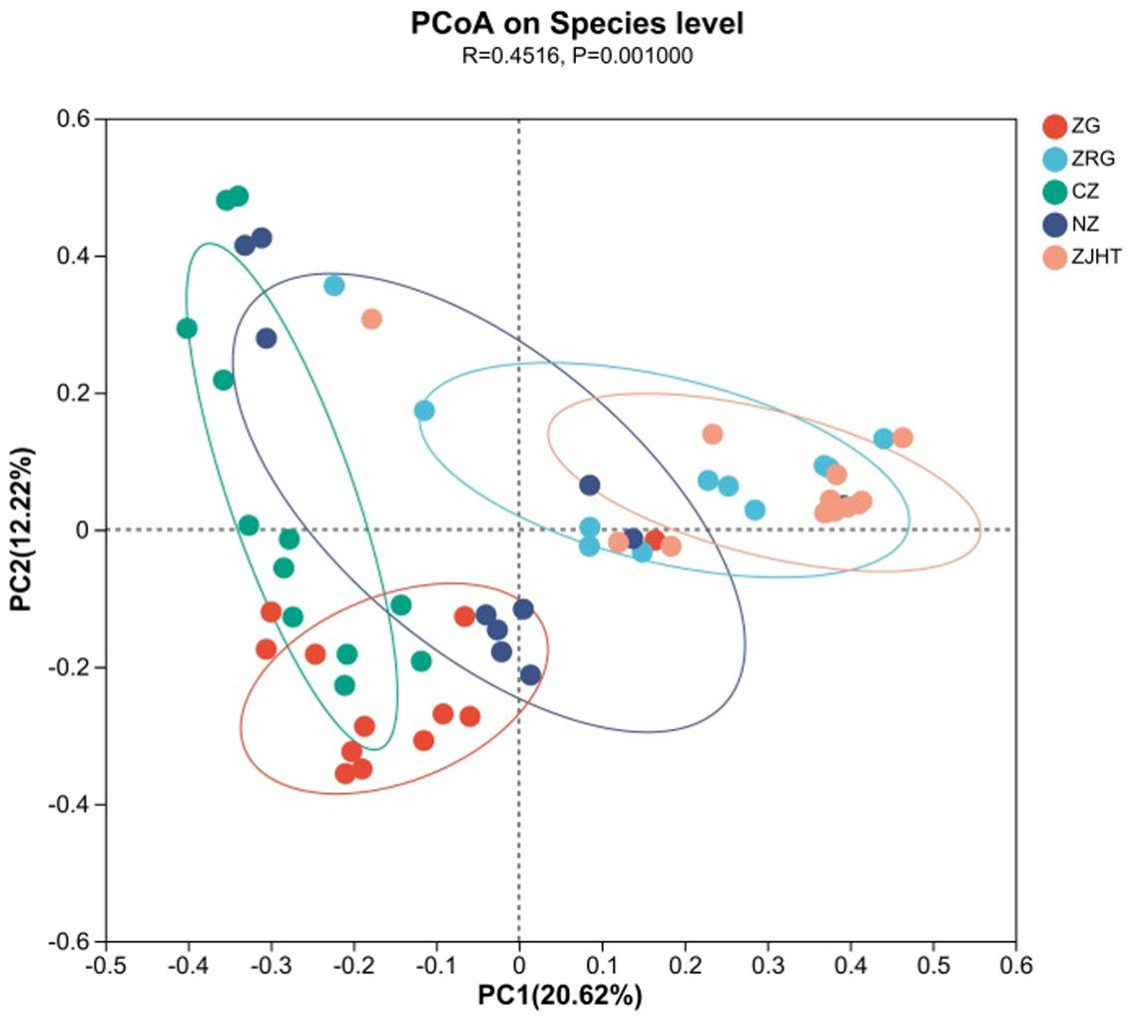
Weighted principal coordinate analysis. ZG represents dried jujube; ZRG represents jujube kernel cake; CZ represents crisp jujube; NZ represents milk date; ZJHT represents jujube with walnut.

Methods such as the weighted PCoA were employed to compare community structures across different jujube products. As illustrated in Figure 2, based on the Bray-Curtis distances in PCoA, the sum of the horizontal and vertical coordinates was 32.84% (20.62% + 12.22%), reflecting a complex fungal community structure that would be difficult to characterize using two principal coordinates (PCs). There were extremely significant differences in the composition of fungal communities among the various samples (*p* < 0.01). It is noteworthy that there was a highly significant difference (*p* < 0.01) in the fungal community composition of the ZRG, CZ, and ZJHT. Similarly, there was a highly significant difference in fungal flora composition between ZG and ZJHT (*p* < 0.01). Meanwhile, significant differences in fungal flora composition were found between ZRG and ZJHT (*p* < 0.01). The CZ samples were observed to be distant in the PC2 direction, indicating the influence of PC2 on the fungal composition of this product. Based on the discrete distribution of sample points, it was also seen that the fungal composition of the CZ was quite different.

### 3.3 Analysis of fungal community structure

**Figure 3:**
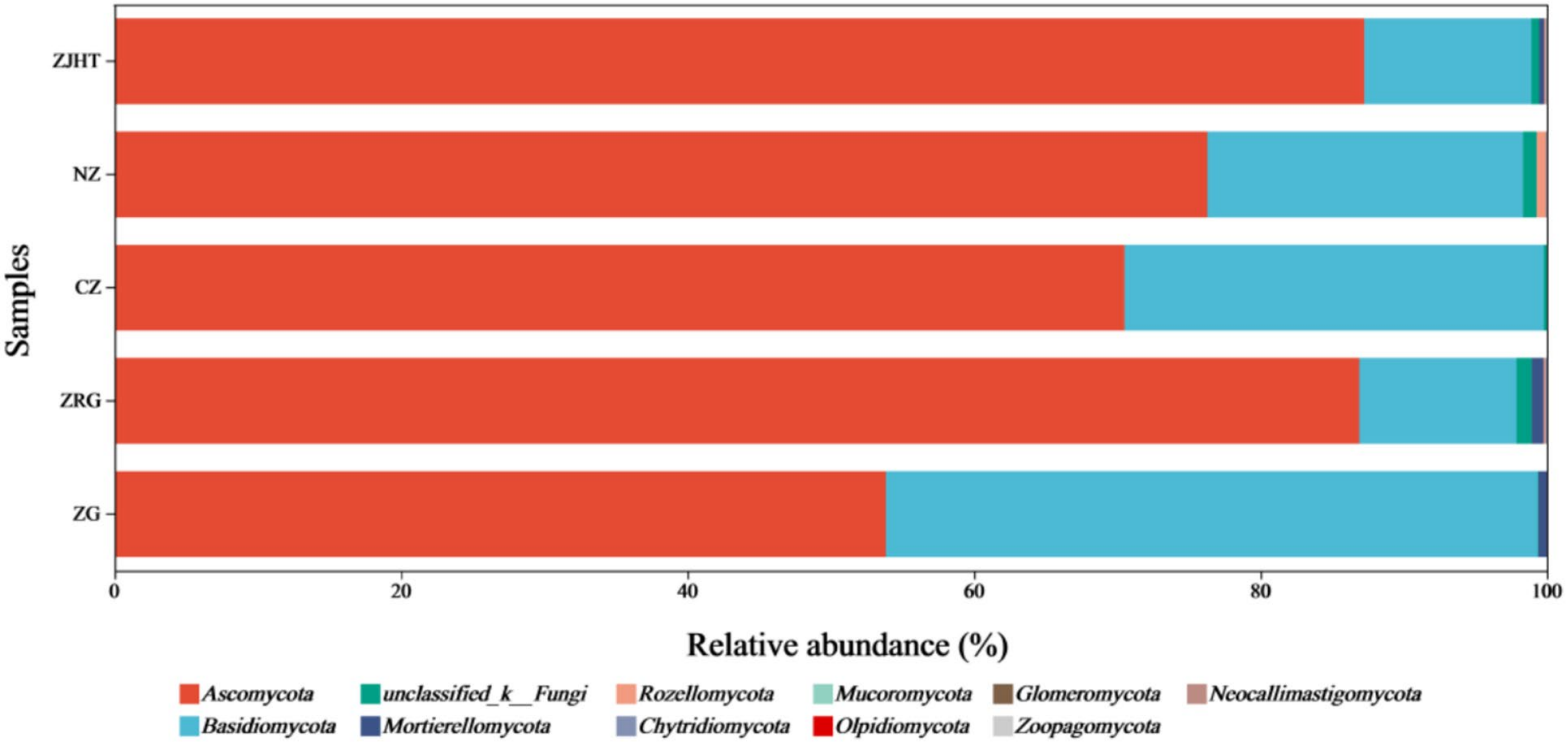
Relative abundance of fungi at the phylum level. ZG represents dried jujube; ZRG represents jujube kernel cake; CZ represents crisp jujube; NZ represents milk date; ZJHT represents jujube with walnut.

**Figure 4:**
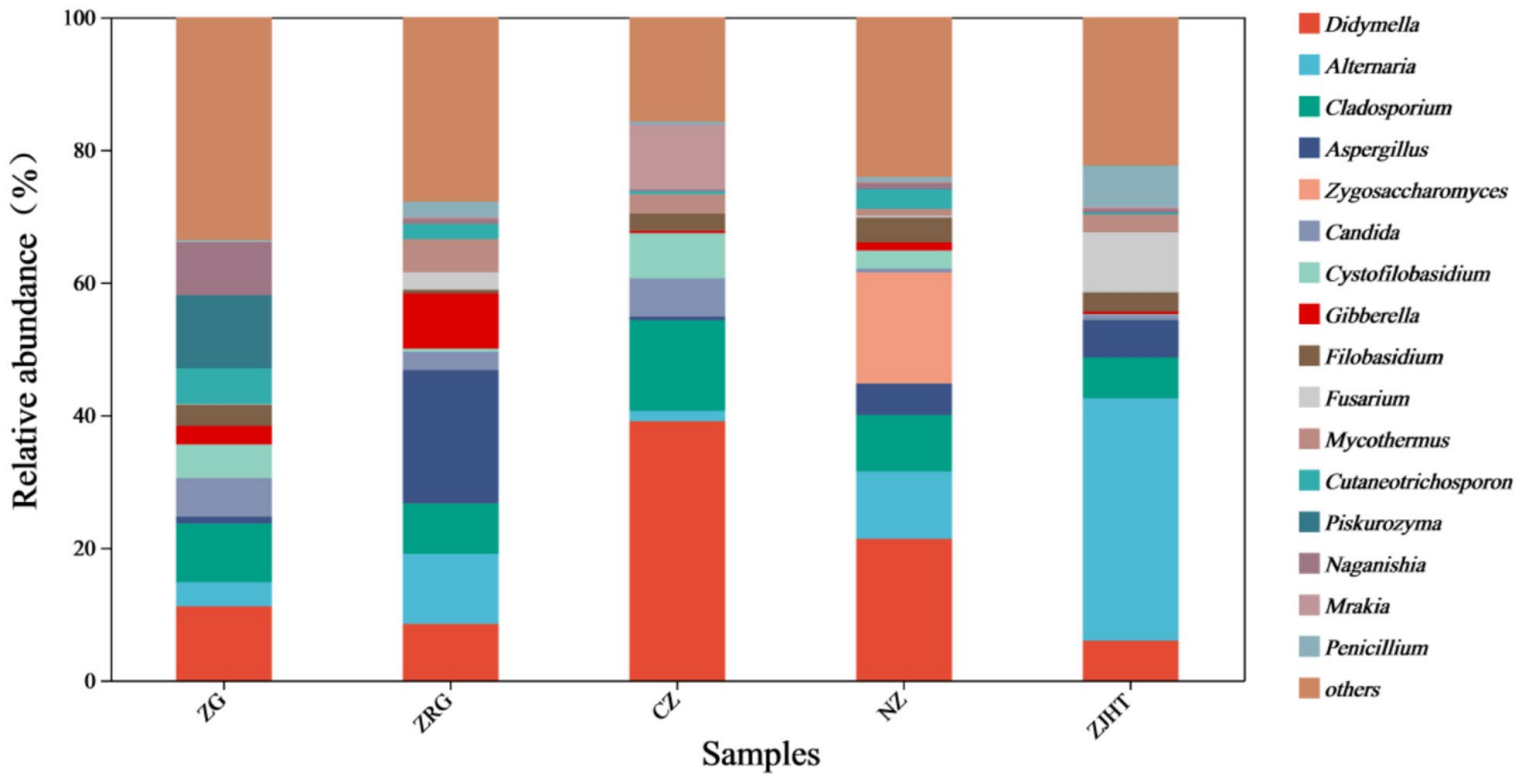
Relative abundance of fungi at the genus level. ZG represents dried jujube; ZRG represents jujube kernel cake; CZ represents crisp jujube; NZ represents milk date; ZJHT represents jujube with walnut.

As depicted in Figure 3, taxonomical classification demonstrated the presence of two fungal phyla in all samples, namely, *Ascomycota* and *Basidiomycota*. *Ascomycota* was the most dominant phylum, accounting for 53.85 to 87.23% of the fungi in all samples. Among all samples, the ZJHT sample has the highest proportion of *Ascomycota* (87.23%). It is worth noting that the ZG sample has the lowest percentage of *Ascomycota* (53.85%), but its *Basidiomycota* proportion (45.50%) is the highest, roughly 4 times that of the ZRG (45.50/10.98) and the ZJHT (45.50/11.67). As reflected in Figure 4, the dominant fungal genera present in product samples such as *Didymella*, *Alternaria*, *Cladosporium*, *Aspergillus*, and *Zygosaccharomyces*, all belonged to the *Ascomycota* phylum. Hence, the dominant fungal genera were consistent with the dominant fungal phyla.

**Figure 5:**
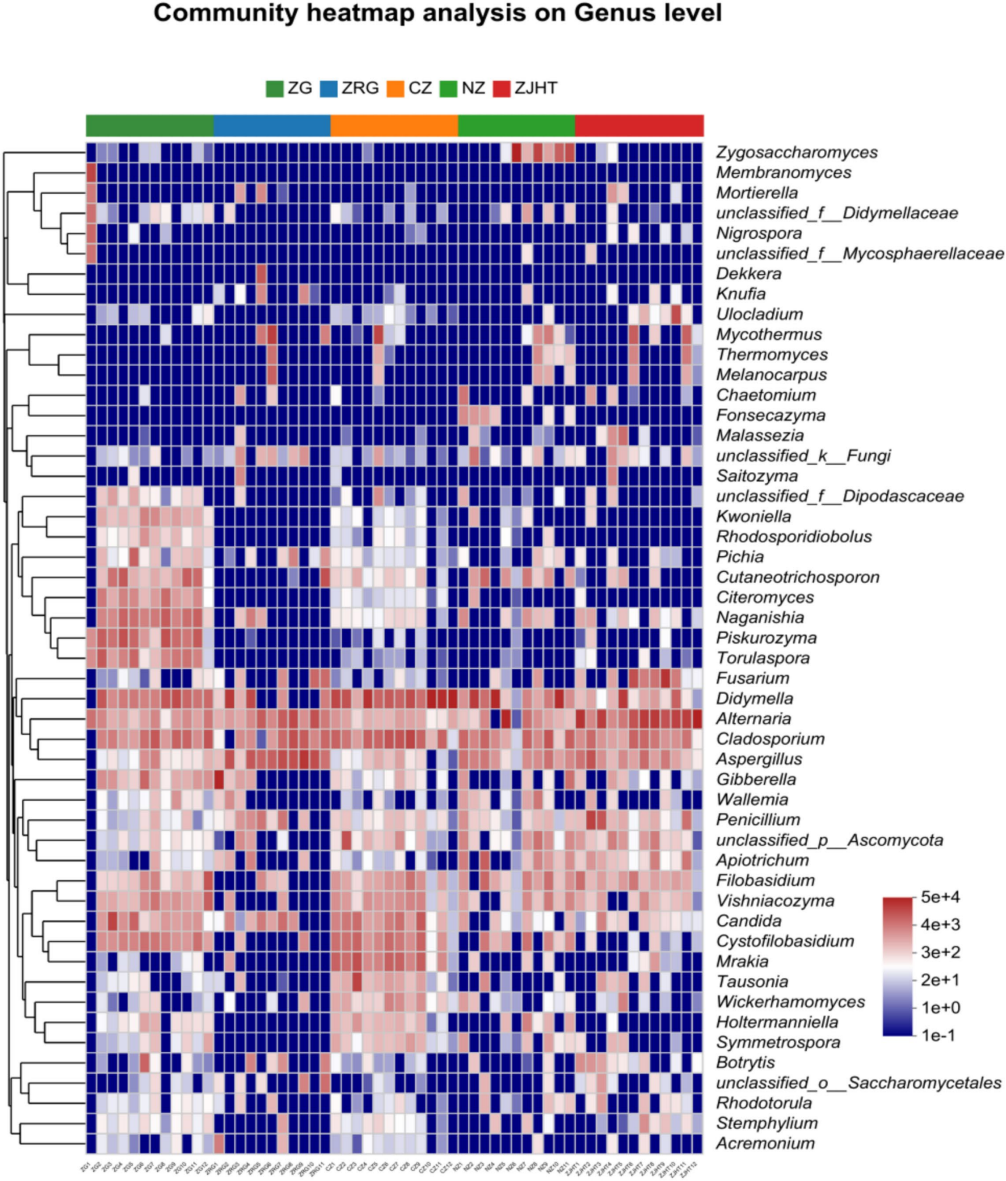
Heatmap of fungal community structure at the genus level. ZG represents dried jujube; ZRG represents jujube kernel cake; CZ represents crisp jujube; NZ represents milk date; ZJHT represents jujube with walnut.

**Figure 6:**
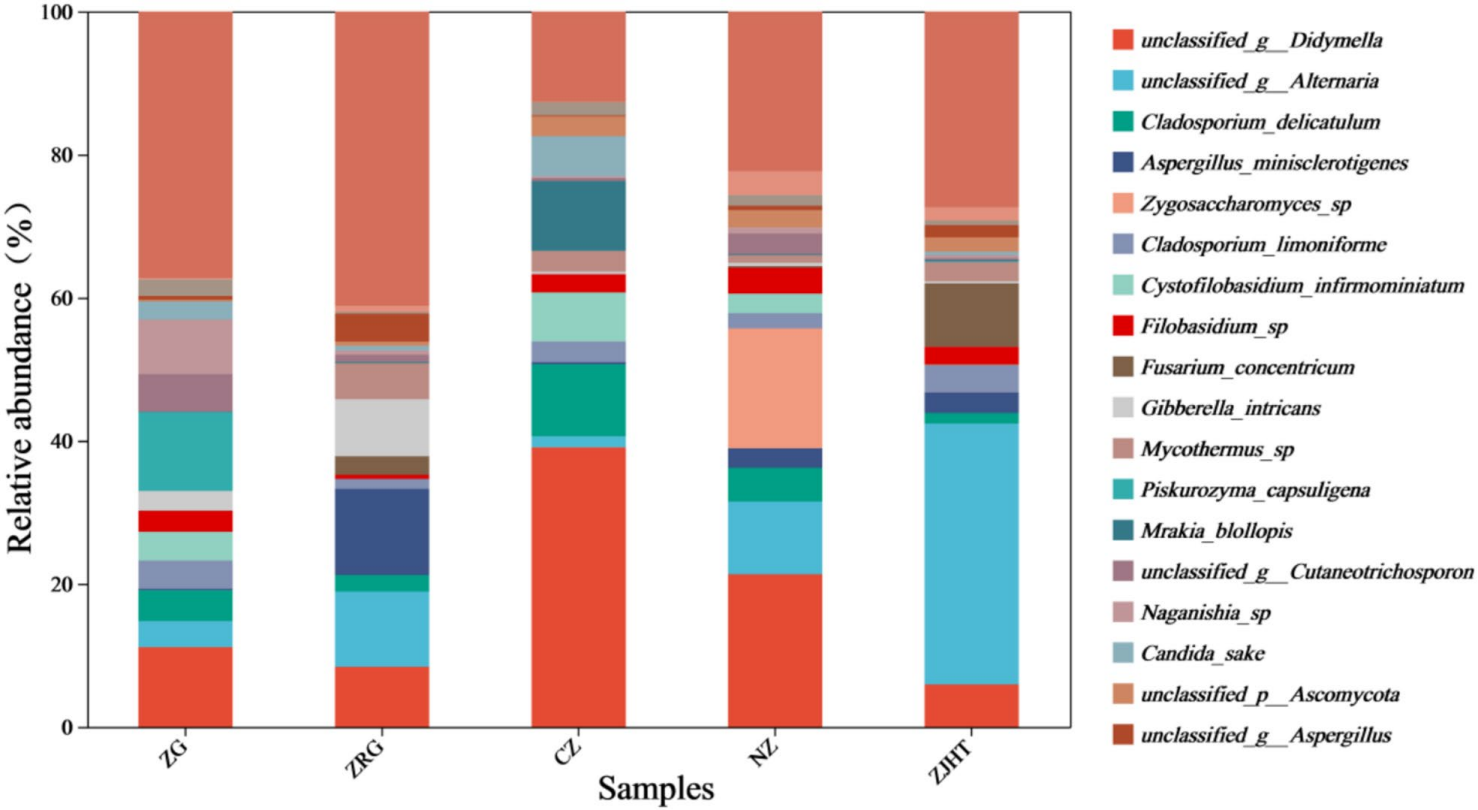
Relative abundance of fungi at the species level. ZG represents dried jujube; ZRG represents jujube kernel cake; CZ represents crisp jujube; NZ represents milk date; ZJHT represents jujube with walnut.

As noted in Figure 5, *Didymella*, *Alternaria*, *Cladosporium*, and *Aspergillus* were detected in all samples with relatively high proportions, indicating they are the dominant fungal strains in the samples. Among all samples, the CZ sample had the highest relative abundance of *Didymella* and *Cladosporium*, the ZJHT sample had the highest relative abundance of *Alternaria*, and the ZRG sample had the highest relative abundance of *Aspergillus*. Additionally, it is important to note that *Fusarium* and *Penicillium* were primarily found in the ZJHT sample, *Candida* was mainly present in the CZ sample, and *Piskurozyma* was predominantly identified in the ZG sample. As shown in Figure 6, the fungal strains that were identified, such as *Cladosporium delicatulum*, *A. minisclerotigenes*, *Fusarium concentricum*, etc. *Alternaria* and *Aspergillus*, were only able to be identified at the genus level, and not species. It is notable that *Zygosaccharomyces* was detected exclusively in the NZ sample, while *Piskurozyma capsuligena* was found only in the ZG sample.

Integrating the aforementioned findings, it could be concluded that *Alternaria*, *Cladosporium*, and *Aspergillus* are the predominant fungal genera in the product samples. Additionally, fungi known to produce mycotoxins, such as *Alternaria* and *Aspergillus*, were detected in most samples, which could potentially affect the quality of processed jujube products.

### 3.4 Isolation and identification of fungi in processed jujube products

A total of 105 fungal strains were isolated from the processed jujube product samples. The molecular identification results of the 105 strains are noted in Supplementary Table S3, and the phylogenetic trees of *Aspergillus* and *Penicillium* are depicted in Supplementary Figures S1, S2.

The number and proportion of fungi across the different processed jujube products are displayed in Table 3. The comprehensive analysis in conjunction with Table 2 indicated that the ZJHT with higher water activity had a higher number of isolated strains (35, 33.33%). In contrast, the CZ with lower water activity also had a considerable number of isolated strains (13, 12.38%). Interestingly, despite the higher water activity of the NZ, the number of isolated strains was relatively low (17, 16.19%).

**Table 3:** The number and proportion of fungal genera in different processed jujube products.

Sample kind	*Penicillium*		*Aspergillus*		*Alternaria*		*Talaromyces*		*Cladosporium*		Total number of strains	Ratio (%)
	Number	Ratio (%)	Number	Ratio (%)	Number	Ratio (%)	Number	Ratio (%)	Number	Ratio (%)		
ZG	1	0.95	9	8.57	0	0.00	2	1.90	1	0.95	13	12.38
ZRG	6	5.71	17	16.19	1	0.95	0	0.00	1	0.95	25	23.81
CZ	2	1.90	6	5.71	1	0.95	4	3.81	2	1.90	15	14.29
NZ	3	2.86	8	7.62	1	0.95	2	1.90	3	2.86	17	16.19
ZJHT	5	4.76	26	24.76	2	1.90	1	0.95	1	0.95	35	33.33
Gross sample	17	16.19	66	62.86	5	4.76	9	8.57	8	7.62	105	100

The ratio columns refer to the ratio of the number of genus − level fungi in the processed red jujube product to the total number of isolated fungi; ZG represents dried jujube; ZRG represents jujube kernel cake; CZ represents crisp jujube; NZ represents milk date; ZJHT represents jujube with walnut.

**Figure 7:**
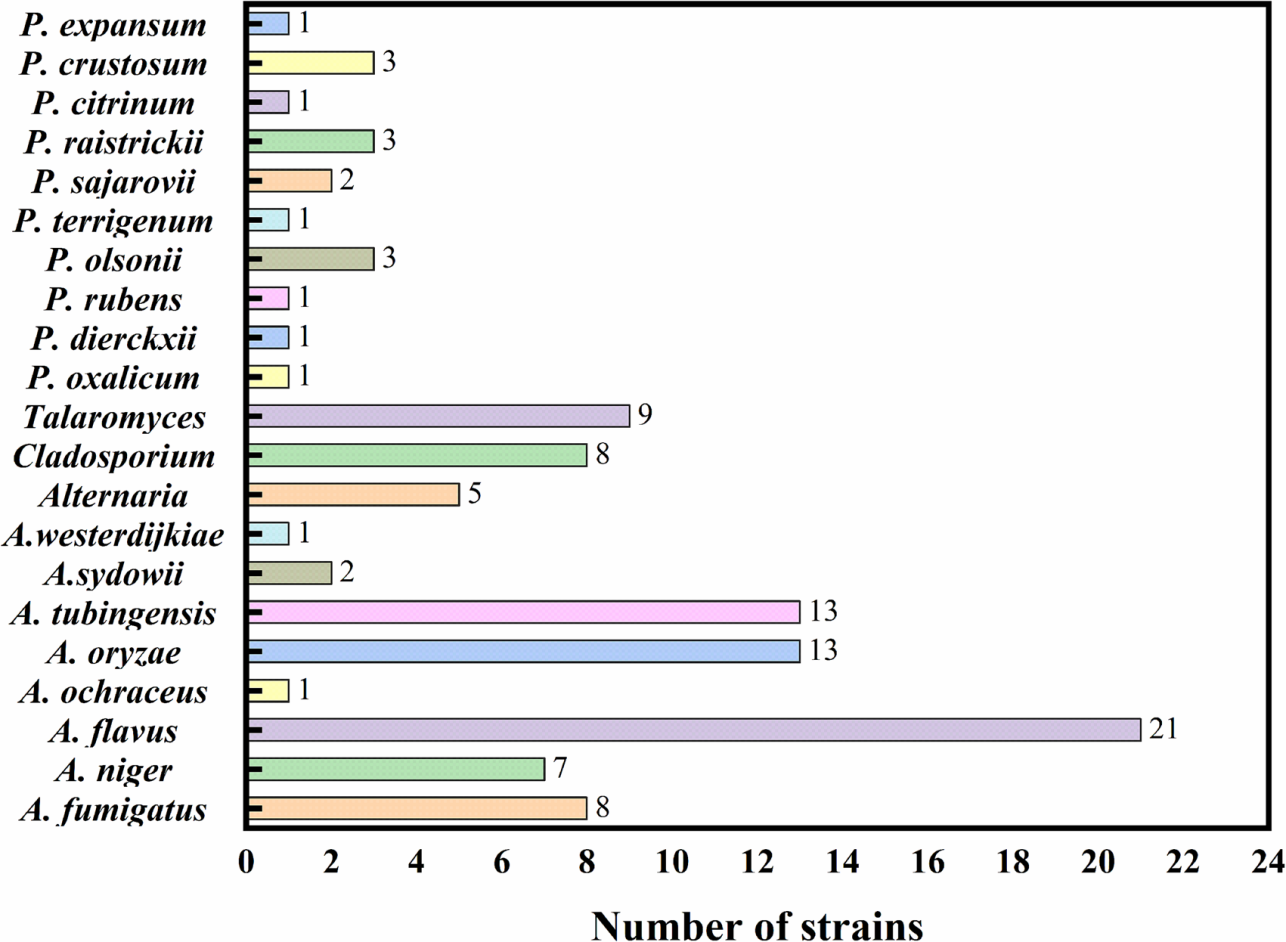
The species and quantity of fungi in processed jujube products.

The species and quantity of fungi isolated from the processed jujube products are illustrated in Figure 7. In the study of processed jujube product samples, a total of 105 fungal strains were isolated and identified. As noted in Table 3, among all the isolated strains, there were 17 strains of the *Penicillium.* Notably, the ZRG had the highest isolation rate of *Penicillium* (5.71%, 6/105), followed by the ZJHT (4.76%, 5/105). Among these *Penicillium*, *P. olsonii*, *P. crustosum,* and *P. raistrickii* were the main species, along with other types such as *P. dierckxii, P. oxalicum, P. rubens, P. terrigenum, P. sajarovii, P. citrinum*, and *P. expansum*. Furthermore, the study also isolated and identified 66 strains of the *Aspergillus*. Among all the product samples, the proportion of *Aspergillus* isolated from ZRG was the highest (16.19%, 17/105), followed by the ZJHT (24.76%, 26/105). Among these *Aspergillus*, *A. fumigatus, A. flavus, A. oryzae, A. niger*, and *A. tubingensis* were the main species, along with other types such as *A. ochraceus, A. sydowii*, and *A. westerdijkiae*. Furthermore, in the samples of NZ, ZRG, ZJHT, and CZ, the 5 strains of *Alternaria* were successfully isolated. At the same time, the 9 strains of *Talaromyces* were isolated from the CZ, the NZ, and the ZG, as well as the 8 strains of *Cladosporium* were primarily sourced from the CZ and the NZ.

### 3.5 Detection results of toxin-producing genes of typical *Aspergillus* spp. in processed jujube products

The results derived from the PCR amplification analysis of the aflatoxin-producing genes of *A. flavus* are shown in Table 4. The seven aflatoxin-producing genes of *A. flavus* tested were *alfQ* (57.14%), *alfP* (52.38%), *aflR* (47.62%), *aflD* (47.62%), *omtA* (42.86%), *ver* (38.10%), and *alfS* (33.33%). In the product samples, a total of 21 *A. flavus* strains were isolated. Especially, five of these strains carried seven aflatoxin-producing genes, and nine strains carried four or more aflatoxin-producing genes. A total of 14 *A. flavus* strains were found to be positive for aflatoxin-producing genes, while the others were negative for the expression of these genes.

**Table 4:** Amplification results of aflatoxin−producing genes of *A. flavus.*

Serial number	Name of strain	Strain origin	Amplification results of aflatoxin−producing genes						
			*aflD*	*aflP*	*alfQ*	*aflR*	*aflS*	*omtA*	*ver*
1	*A. flavus*	ZG	+	+	−	+	−	−	−
2	*A. flavus*	ZRG	−	+	+	−	−	−	+
3	*A. flavus*	ZG	−	−	−	−	−	−	−
4	*A. flavus*	CZ	+	+	+	+	+	+	+
5	*A. flavus*	ZRG	+	+	+	+	+	+	+
6	*A. flavus*	ZJHT	−	−	−	−	−	−	−
7	*A. flavus*	NZ	+	+	+	+	+	+	+
8	*A. flavus*	ZG	+	+	+	+	+	+	+
9	*A. flavus*	CZ	−	+	−	−	−	−	−
10	*A. flavus*	ZRG	−	−	−	−	−	−	−
11	*A. flavus*	ZJHT	+	+	+	+	+	+	+
12	*A. flavus*	ZRG	−	−	−	−	−	−	−
13	*A. flavus*	ZJHT	−	−	+	−	+	−	+
14	*A. flavus*	ZJHT	−	−	−	−	−	−	−
15	*A. flavus*	NZ	−	−	−	−	−	−	−
16	*A. flavus*	ZJHT	+	−	+	+	−	+	−
17	*A. flavus*	ZJHT	+	+	+	+	−	−	+
18	*A. flavus*	ZJHT	−	−	−	−	−	−	−
19	*A. flavus*	ZRG	+	+	+	+	+	+	−
20	*A. flavus*	ZJHT	−	−	+	−	−	+	−
21	*A. flavus*	ZJHT	+	+	+	+	−	+	−

ZG represents dried jujube; ZRG represents jujube kernel cake; CZ represents crisp jujube; NZ represents milk date; ZJHT represents jujube with walnut.

The results of the PCR amplification analysis of the ochratoxin A-producing genes of *A. niger, A. tubingensis* and *A. ochraceus* are indicated in Table 5. The seven ochratoxin A-producing genes of *A. niger*, *A. tubingensis,* and *A. ochraceus* tested were *pks* (61.90%), *nrps* (57.14%), *otaA* (52.38%), *otaD* (52.38%), *otaB* (47.62%), *AcLaeA* (28.57%), and *otaC* (28.57%). A total of 21 strains (7 *A. niger*, 13 *A. tubingensis,* and 1 *A. ochraceus*) were isolated from the samples. It is very noteworthy that three of these strains carried all 7 ochratoxin A-producting genes and 11 strains carried 4 or more ochratoxin A-producting genes. In total, the 13 strains were positive for the expression of ochratoxin A-producing genes, while the others were negative for their expression. Detailed toxin production traits in these fungi need to be verified through further experiments.

**Table 5:** Amplification results of ochratoxin A − producing genes of *A. niger, A. tabingensis*, and *A. ochraceus.*

Serial number	Name of strain	Strain origin	Amplification results of ochratoxin A − producing genes						
			*AcLaeA*	*otaA*	*otaB*	*otaC*	*otaD*	*pks*	*nrps*
1	*A. niger*	ZRG	−	+	−	−	+	+	+
2	*A. niger*	CZ	−	−	−	−	−	−	−
3	*A. niger*	ZG	−	−	−	−	−	−	−
4	*A. niger*	ZRG	+	+	+	−	+	+	+
5	*A. niger*	NZ	+	−	+	−	−	+	+
6	*A. tubingensis*	ZG	+	+	−	−	+	+	+
7	*A. tubingensis*	ZRG	−	−	−	−	−	−	−
8	*A. niger*	ZG	−	−	−	−	−	−	−
9	*A. tubingensis*	ZJHT	+	+	+	+	+	+	+
10	*A. niger*	ZRG	−	−	−	−	−	−	−
11	*A. tubingensis*	ZJHT	−	+	+	−	+	+	+
12	*A. tubingensis*	ZJHT	−	−	−	−	+	+	+
13	*A. tubingensis*	ZJHT	−	+	+	−	+	+	+
14	*A. tubingensis*	ZRG	−	+	+	+	+	+	+
15	*A. tubingensis*	ZRG	+	+	+	+	+	+	+
16	*A. tubingensis*	ZJHT	−	+	+	+	+	+	+
17	*A. tubingensis*	CZ	−	−	−	−	−	−	−
18	*A. ochraceus*	ZJHT	+	+	+	+	+	+	+
19	*A. tubingensis*	ZRG	−	−	−	−	−	−	−
20	*A. tubingensis*	ZJHT	−	+	+	+	−	+	+
21	*A. tubingensis*	NZ	−	−	−	−	−	−	−

ZG represents dried jujube; ZRG represents jujube kernel cake; CZ represents crisp jujube; NZ represents milk date; ZJHT represents jujube with walnut.

### 3.6 Production of mycotoxins by typical *Aspergillus* spp. in processed jujube products

As shown in Table 6, a total of 21 strains of *A. flavus* were isolated from all product samples. It notably showed that 10 of these strains were capable of producing aflatoxin. Among them, we found that only one strain produced AFB_1_, but two strains produced both AFB_1_ and AFB_2_. Among these 10 strains, the levels of AFT, AFB_1_, AFB_2_, AFM_1_, and AFM_2_ were in the ranges of 19.3862–21.7583 μg/L, 6.5309–11.0411 μg/L, 0–15.4407 μg/L, 0–5.6354 μg/L, 0–6.0545 μg/L, with the average ranges of 20.1449 μg/L, 8.8021 μg/L, 9.8160 μg/L, 3.1780 μg/L and 3.5710 μg/L, respectively. It could be concluded that *A. flavus* strains in processed jujube products have the strongest ability to produce AFB_1_ toxin. As shown in Table 7, the 7 strains of *A. niger*, 13 strains of *A. tubingensis*, and 1 strain of *A. ochraceus* were isolated from processed jujube products, of which 8 strains produced OTA. OTA content ranged from 5.2019 to 18.5207 μg/L, with a mean value of 8.3345 μg/L. From the aforementioned results, it could be inferred that the presence of *Aspergillus* with potent mycotoxin production capabilities can be isolated from product samples, which may pose a potential safety concern affecting the processed jujube products.

**Table 6:** Aflatoxin content and types produced by 21 strains of *A. flavus.*

Name of strain	Strain origin	AFT (μg/L)	AFB_1_ (μg/L)	AFB_2_ (μg/L)	AFM_1_ (μg/L)	AFM_2_ (μg/L)
*A. flavus*	ZG	19.4271 ± 0.0053	6.5309 ± 0.0245	11.6912 ± 0.0166	2.9530 ± 0.0490	4.1815 ± 0.0180
*A. flavus*	ZRG	−	−	−	−	−
*A. flavus*	ZG	−	−	−	−	−
*A. flavus*	CZ	19.8077 ± 0.0059	8.3042 ± 0.0095	10.7021 ± 0.0122	5.6354 ± 0.0588	5.1991 ± 0.0488
*A. flavus*	ZRG	19.4390 ± 0.0048	8.5577 ± 0.0188	8.7941 ± 0.0238	−	−
*A. flavus*	ZJHT	−	−	−	−	−
*A. flavus*	NZ	21.7583 ± 0.0004	9.1858 ± 0.0086	13.7410 ± 0.0374	4.8912 ± 0.0137	5.4986 ± 0.0068
*A. flavus*	ZG	19.9410 ± 0.0058	10.1383 ± 0.0280	8.6137 ± 0.0335	−	−
*A. flavus*	CZ	−	−	−	−	−
*A. flavus*	ZRG	−	−	−	−	−
*A. flavus*	ZJHT	21.7329 ± 0.0008	10.9148 ± 0.0434	11.4122 ± 0.0432	3.6209 ± 0.0090	5.1143 ± 0.0105
*A. flavus*	ZRG	−	−	−	−	−
*A. flavus*	ZJHT	−	−	−	−	−
*A. flavus*	ZJHT	−	−	−	−	−
*A. flavus*	NZ	−	−	−	−	−
*A. flavus*	ZJHT	20.1880 ± 0.0031	8.7333 ± 0.0869	15.4407 ± 0.0267	4.4796 ± 0.0089	4.7947 ± 0.0193
*A. flavus*	ZJHT	19.3862 ± 0.0053	6.7583 ± 0.0238	10.0495 ± 0.0129	5.4882 ± 0.0272	4.8830 ± 0.0209
*A. flavus*	ZJHT	−	−	−	−	−
*A. flavus*	ZRG	20.0273 ± 0.0043	7.8566 ± 0.0323	7.7156 ± 0.0061	4.7098 ± 0.1056	6.0545 ± 0.0126
*A. flavus*	ZJHT	−	−	−	−	−
*A. flavus*	ZJHT	19.7410 ± 0.0020	11.0411 ± 0.0382	−	−	−

ZG represents dried jujube; ZRG represents jujube kernel cake; CZ represents crisp jujube; NZ represents milk date; ZJHT represents jujube with walnut.

**Table 7:** OTA content by 7 strains of *A. niger*, 13 strains of *A. tubingensis*, and 1 strain of *A. ochraceus*.

Name of strain	Strain origin	OTA (μg/L)
*A. niger*	ZRG	−
*A. niger*	CZ	9.2742 ± 0.0220
*A. niger*	ZG	−
*A. niger*	ZRG	−
*A. niger*	NZ	6.9835 ± 0.0639
*A. tubingensis*	ZG	−
*A. tubingensis*	ZRG	−
*A. niger*	ZG	−
*A. tubingensis*	ZJHT	−
*A. tubingensis*	ZRG	5.2019 ± 0.0242
*A. niger*	ZJHT	−
*A. tubingensis*	ZJHT	7.0344 ± 0.0206
*A. tubingensis*	ZJHT	−
*A. tubingensis*	ZRG	−
*A. tubingensis*	ZRG	−
*A. tubingensis*	ZJHT	−
*A. tubingensis*	CZ	5.8803 ± 0.0698
*A. tubingensis*	ZJHT	−
*A. tubingensis*	ZRG	7.0712 ± 0.0537
*A. ochraceus*	ZJHT	18.5207 ± 0.0487
*A. tubingensis*	NZ	6.7099 ± 0.0920

ZG represents dried jujube; ZRG represents jujube kernel cake; CZ represents crisp jujube; NZ represents milk date; ZJHT represents jujube with walnut.

## 4 Discussion

### 4.1 Isolation and diversity of fungi from processed jujube products

The presence of fungi in jujube and processed jujube products has been frequently reported worldwide in recent years. *Aspergillus*, *Alternaria*, *Cladosporium*, and *Penicillium* were the most prevailing genera detected (Okayo et al., 2020; Sajidamu et al., 2018; Ghazi-Yaker et al., 2022; Tournas et al., 2015). This study also reached similar conclusions. All processed jujube product samples exhibited fungal contamination. At the phylum level, *Ascomycota* and *Basidiomycota* were dominant. The isolated strains were primarily from the *Aspergillus*, followed by *Penicillium.* In particular, although the NZ sample has a high water activity, it has a lower total colony count. Mizzi et al. (2020) discovered that the addition of auxiliary materials during the processing could affect the growth of fungi. Due to the potential inhibitory effect of high sugar content on fungal growth, it could be the reason for the lower total number of colonies in the NZ. Noteworthily, the most frequently isolated strains within the *Aspergillus* were *A. flavus*, *A. niger*, and *A. tubingensis*. Many of these fungi are capable of producing mycotoxins, such as aflatoxins and ochratoxins (Fouad et al., 2019; Zhao et al., 2020). These mycotoxins could produced neurotoxic, carcinogenic, and other toxic effects, posing a threat to consumer health even at low levels (Xin et al., 2023; Samuel et al., 2021; Rocha et al., 2023).

Traditional culture techniques, limited by the cultivability of microorganisms (e.g., some fungi are difficult to grow or remain dormant on artificial culture media), fail to comprehensively analyze the true composition of fungal communities in processed jujube products, leading to insufficient analysis of fungal diversity within samples (Daniel, 2004). In contrast, high-throughput sequencing technology, based on culture-independent metagenomics strategies, can deeply analyze the ITS sequence information of all fungi in samples, significantly improving the sensitivity of species detection (e.g., detecting low-abundance or hard-to-culture microbial communities). In this study, the application of this technology revealed variations in fungal composition among different product samples. In CZ samples, the dominant fungi were *Didymella* (39.07%) and *Cladosporium* (13.68%), with the highest relative abundance. This may be due to the fact that *Didymella* is a fungus tolerant to high osmotic pressure, and *Cladosporium* spores have strong stress resistance and a strong ability to withstand desiccation (Correia et al., 2024; Duran et al., 2010). In ZJHT samples, *Alternaria* had the highest relative abundance (36.56%). This could be attributed to two reasons. On the one hand, the high water activity of jujube and walnut products, combined with *Alternaria*’s ability to adapt to adverse conditions, may promote its growth (Wang et al., 2024). On the other hand, the addition of walnuts during processing may contribute to the prevalence of *Alternaria*, as it is a dominant fungus in both jujube and walnut (Wei et al., 2020). Additionally, our results indicate significant differences in fungal composition among the samples (*p* < 0.01). On the one hand, the processing of jujube products (such as frying and drying) might alter the structure of the fungi. On the other hand, the addition of auxiliary materials during the product processing might affect the fungal communities present in the products (Isabel Galvan et al., 2022). The two reasons mentioned above might account for the differences in communities between all samples.

### 4.2 Analysis of toxin production in processed jujube products

In recent years, the contamination of mycotoxins in processed jujube products has garnered increasing global attention. A study on the fungal and mycotoxin contamination of dried dates showed that 2.9% detection rate of AFB_1_ in dried dates, with levels below 700 μg/kg (Wang et al., 2018). For instance, Han et al. (2016) assessed the presence of mycotoxins in dried fruits such as red jujube and detected OTA in 22.5% of jujube products. The detected levels of OTA ranged from 0.5 to 61.4 μg/L. In another investigation of ochratoxin A contamination in dried fruits such as jujubes collected from the United States, the results demonstrated that the OTA detection rate of dates was 2%, in which the OTA content was 0.39 μg/L on average (Palumbo et al., 2015). Similarly, in this study, strains producing aflatoxins and OTA were also detected.

In the present research, among the strains isolated from processed date products, 10 aflatoxin-producing strains and 8 ochratoxin A-producing strains were identified. Among the aflatoxins, the production level of AFB_1_ was the highest, ranging from 6.5309 to 11.0411 μg/L. Additionally, the production levels of OTA ranged from 5.2019 to 18.5207 μg/L. Remarkably, aflatoxins are the most prevalent and toxic of nearly 400 mycotoxins identified in date samples (Deng et al., 2021). Additionally, ochratoxins are the most widely studied mycotoxins globally after aflatoxins (Agriopoulou et al., 2020). Therefore, in this study, the isolation of fungi with strong mycotoxin-producing capabilities from processed jujube products, along with the high toxicity of the fungal toxins, poses a significant potential threat to consumer health.

The European Union has established stringent standards for the levels of major mycotoxins in nuts, dried fruits, and their products manufactured for direct consumption. For these dried fruits intended for direct consumption, the maximum level of AFB1 is set at 2 μg/kg, and the combined maximum level of AFB_1_ + B_2_ + G_1_ + G_2_ is set at 4 μg/kg. For processed nuts and dried fruits (e.g., those subjected to frying), the maximum permissible level of AFB_1_ is 5 μg/kg, and the limit for AFT is 10 μg/kg (Li et al., 2017). Additionally, for products containing oilseeds, nuts, and/or dried fruits (excluding raisins and figs), the maximum level of OTA is set at 2.0 μg/kg (Commission Regulation (EU), 2022). There are no specific standards for aflatoxins and ochratoxins for processed jujube products. To ensure food safety, ready-to-eat jujube products should adhere to strict standards and testing procedures. The presence of toxigenic fungi in jujube products on the market could pose a threat to human health, as the toxins produced by these fungi are difficult to decompose and are not easily removed during the processing. In processed jujube products, contaminating fungi and mycotoxins are primarily introduced through the raw jujube materials. Furthermore, controlling conditions such as water activity during processing and storage is also crucial.

Controlling fungi and mycotoxins in jujube raw materials, biological control is a widely used method currently. This method involves the use of biological agents such as bacteria (*Bacillus, Pseudomonas*, etc.) and fungi (*Yeast, Trichoderma, Aspergillus*, etc.) and biodegradable compounds such as shell glycan and oligosaccharides to not only control the growth of fungi but also reduce mycotoxin production or degrade any produced mycotoxins (Shang et al., 2019; Lei et al., 2022; Liu et al., 2023). Jujubes are the raw materials for processed dates, and controlling the quality of jujubes is very important (Bukhari et al., 2023). Additionally, water activity is an important factor affecting fungal growth and mycotoxin production (Dong et al., 2023). Therefore, in addition to controlling the quality of the raw material for processed jujube products, it is also important to vacuum package these products to reduce their water activity.

## 5 Conclusion

In this study, the fungal diversity and mycotoxin production by typical *Aspergillu*s strains in processed jujube products were analyzed. Traditional microbial isolation and sequencing of ITS and other regions identified 105 fungal strains, with dominant genera including *Aspergillu*s, *Cladosporium*, and *Penicillium*. High-throughput sequencing revealed that the dominant phyla were *Ascomycota* and *Basidiomycota*, with *Alternaria*, *Didymella*, *Cladosporium*, and *Aspergillus* being the prevalent genera. The study found that the aflatoxin-producing strains isolated from processed jujube products had the highest capability to produce AFB_1_, reaching a maximum of 11.0411 μg/L, and the maximum production of AFT was 21.7583 μg/L. The OTA content produced by *A. niger*, *A. tubingensis*, and *A. ochraceus* strains isolated from the product samples ranged from 5.2019 to 18.5207 μg/L. Potential mycotoxin-producing fungi were identified in the processed jujube products. Although the total fungal count was below the Chinese national standard limits, the toxigenic *Aspergillus* strains exhibited strong toxin production capabilities, posing a significant potential risk of mycotoxins and threatening consumer health. This research provides valuable insights into the fungal contamination in processed jujube products. To reduce fungal contamination and toxin risks, guidelines should be established, including the optimization of jujube raw material selection and storage conditions. This study was limited in sample size and region, future research should expand the sample range and delve into contamination factors to protect consumer health.

## Data Availability

The original contributions presented in the study are included in the article/Supplementary material, further inquiries can be directed to the corresponding authors.

## Footnotes

^1^ https://ccb.jhu.edu/software/FLASH/index.Shtml

## Supplementary material

SUPPLEMENTARY FIGURE S1 The phylogenetic tree of *Penicillium* spp. Strains in processed jujube product.

SUPPLEMENTARY FIGURE S2 The phylogenetic tree *Aspergillus* spp. Strains in processed jujube product.

SUPPLEMENTARY FIGURE S3 Distribution of core fungal species in processed jujube products. ZG represents dried jujube; ZRG represents jujube kernel cake; CZ represents crisp jujube; NZ represents milk date; ZJHT represents jujube with walnut.
