# Supplementary figures and images for: Analysis of fungal diversity in processed jujube products and the production of mycotoxins by typical toxigenic Aspergillus spp
Source: Front Microbiol. 2025 Mar 26;16:1499686. doi: 10.3389/fmicb.2025.1499686 (PMC11978838; doi:10.3389/fmicb.2025.1499686)

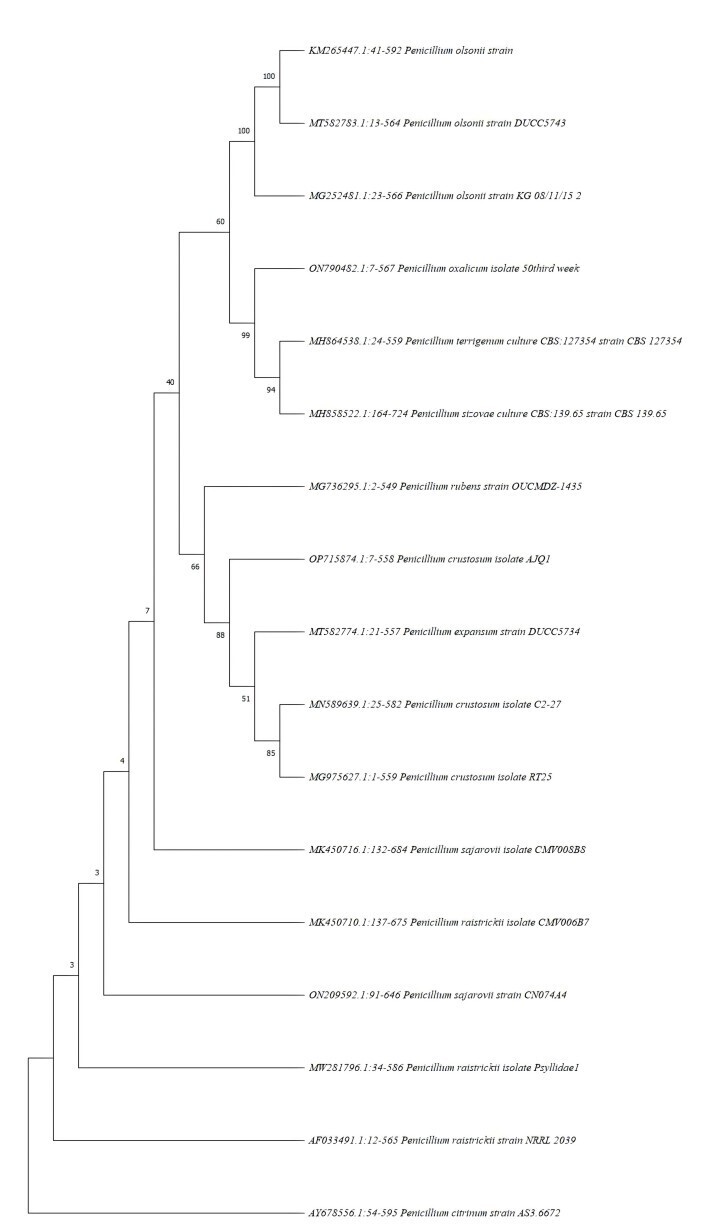

Supplement: SUPPLEMENTARY FIGURE S1 — The phylogenetic tree of Penicillium spp. Strains in processed jujube product. [file Image_1.jpeg]

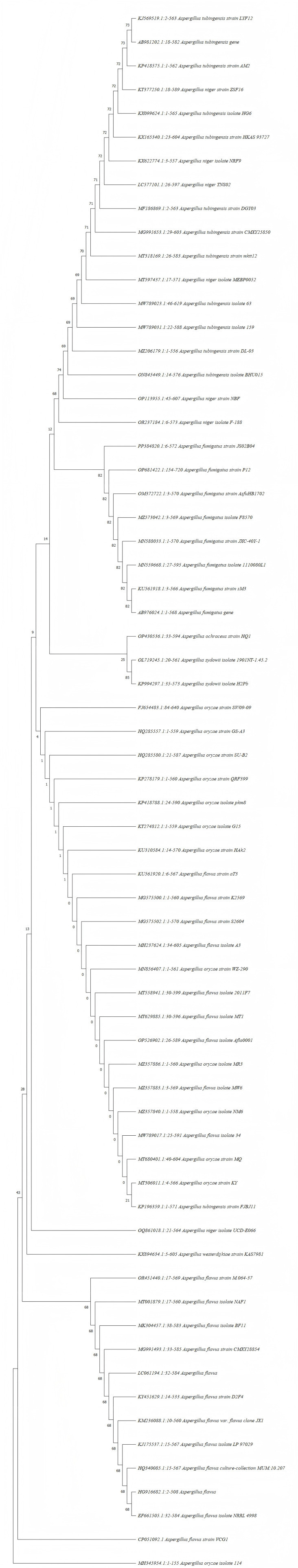

Supplement: SUPPLEMENTARY FIGURE S2 — The phylogenetic tree Aspergillus spp. Strains in processed jujube product. [file Image_2.jpeg]

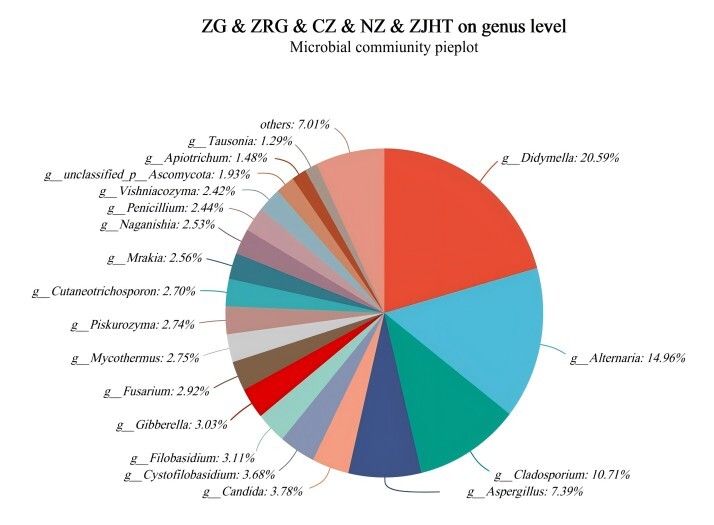

Supplement: SUPPLEMENTARY FIGURE S3 — Distribution of core fungal species in processed jujube products. ZG represents dried jujube; ZRG represents jujube kernel cake; CZ represents crisp jujube; NZ represents milk date; ZJHT represents jujube with walnut. [file Image_3.jpeg]

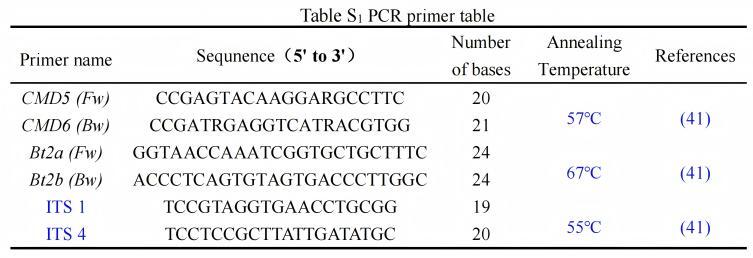

Supplement: Supplementary file 4 [file Image_4.jpeg]

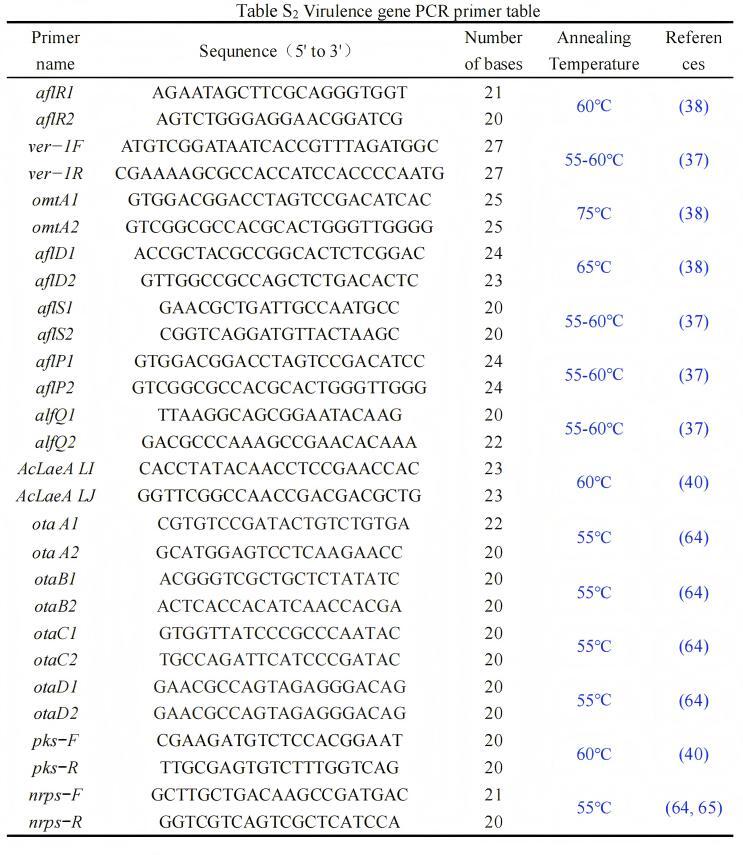

Supplement: Supplementary file 5 [file Image_5.jpeg]

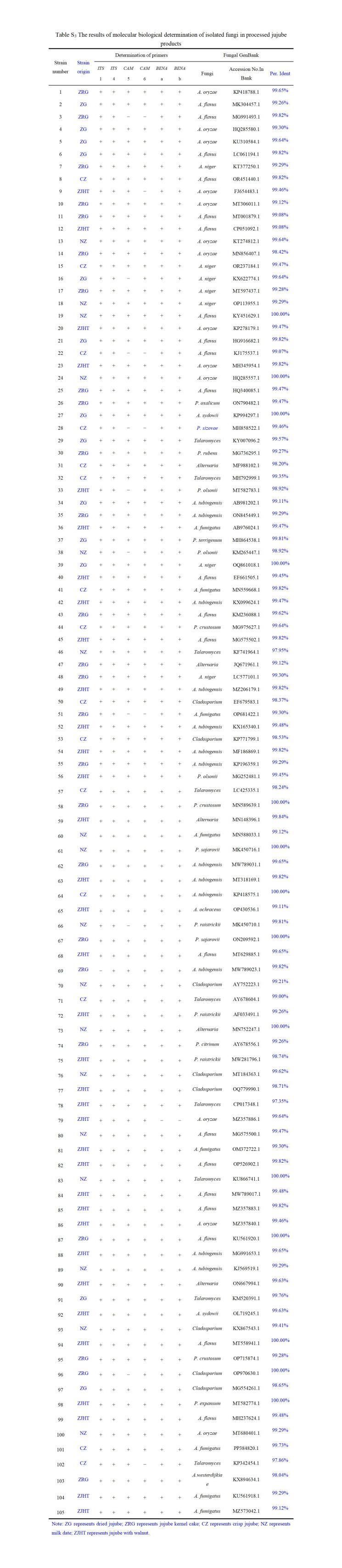

Supplement: Supplementary file 6 [file Image_6.jpeg]
